# Supplementary material for: MetaRibo-Seq measures translation in microbiomes
Source: Nat Commun. 2020 Jun 29;11:3268. doi: 10.1038/s41467-020-17081-z (PMC7324362; doi:10.1038/s41467-020-17081-z)
Supplement: Supplementary file 10 — Supplementary Data 7 [file 41467_2020_17081_MOESM10_ESM.zip › File2/Confidence_High_Taxonomy/41581_out.krona.html]

Javascript must be enabled to view this page.

members
magnitude
magnitudeUnassigned
count
unassigned
taxon
rank

41581\_out

3

3
2
superkingdom

phylum
1239
3

3
186801
class

2
186802
order

541000
1
family


SRS044535\_contig\_number\_1783
species
1898205
1

family
1
216572

459786
1
genus


SRS077392\_contig\_number\_20904
species
1897011
1

1
2044939
species

SRS892882\_contig\_number\_contig-100\_10480.10481
